# Investigation of Extrusion Parameters Affecting 3D Print Performance of Wheat Flour and Citrus Pectin Dough

**DOI:** 10.3390/foods15193361

**Published:** 2026-09-22

**Authors:** Jinjie Luo, Guofeng Xia, Anping Ji, Lizi Liu, Zhenghua Deng, Bin Nie

**Affiliations:** Department of Mechanical Engineering, Chongqing Sanxia University of Science and Techonlogy, Chongqing 404000, China

**Keywords:** three-dimensional food printing, food shaping, food ink, food printing efficiency

## Abstract

In this study, three-dimensional (3D) printed dough samples were prepared using wheat flour powder and citrus pectin. Dough formulations supplemented with 0.2 g, 0.5 g, and 0.7 g citrus pectin exhibited superior printing performance. The optimal values of compressive pressure, nozzle travel speed, nozzle bore diameter, and infill rate for superior printing performance were 350 kPa, 15 mm/s, 1 mm, and 30%, respectively. A compressive pressure of 350 kPa produced the optimal printing performance of samples C and H. The highest printing quality (4.52 ± 0.14) was achieved with a nozzle speed of 15 mm/s, exhibiting uniform layer thickness, clear outlines, and excellent stacking performance. The highest score was observed in sample C (4.45 ± 0.18), indicating superior printing performance. When the nozzle speed was 15 mm/s, the printed samples showed the highest printing quality (4.52 ± 0.14), with uniform layer thickness, clear outlines, and excellent stacking performance. At a filling rate of 30%, the overall appearance and structural integrity of the printed samples, as well as the number and size of internal voids, closely matched the designed structure. Texture profile analysis indicated that pectin acts as a modifier that alters the internal structure of dough and that 3D printing technology may improve the sensory properties of dough products to a certain extent. Therefore, the addition of pectin to the dough and the use of 3D printing technology not only improved the internal structure of the dough but also enhanced product acceptability and provided general insights into the relationship between printing parameters, shape stability, and the properties of food ink materials. Finally, 3D printing technology provided general insights into the relationship between printing parameters, shape stability, and food ink material properties.

## 1. Introduction

Pectin, a natural dietary fiber, is widely present in terrestrial plant cell walls, with citrus serving as the primary source of commercial pectin [1]. As a natural and healthy food hydrocolloid, pectin is widely used in the food industry. Presently, pectin is mainly used as a gelling agent, thickener, and emulsifier in the manufacturing of fruit products, jelly, soft candy, and other foods, with typical addition levels ranging from 0.05 to 5 wt%. Pectin promotes gastrointestinal motility and nutrient absorption [2]. In food products, pectin helps maintain flavor, promotes fat emulsification, and enhances structural stability. Pectin has also been used in baked products and edible films as a fat substitute to retard crumb staling in cakes. Min et al. [3] replaced shortening with pectin, resulting in cookies with a small diameter, tender texture, and light color. As sugar alternatives for cake batter and finished cakes, ultrasound-processed pectins were applied by Chan et al. [4]. Pectin with prolonged sonication times and higher ultrasound amplitudes decreased batter density, thereby increasing cake volume and producing a less dense crumb structure.

As an emerging intelligent manufacturing technology, three-dimensional (3D) printing constructs products layer by layer based on 3D digital model data. Compared with traditional manufacturing methods, 3D printing offers advantages, such as reduced material waste and low production costs, making its application across various manufacturing industries feasible [5]. Within food processing, this technology can meet the demand for personalized food appearance and healthy dietary options among different consumers. One of the most prominent features of food 3D printing is its ability to personalize food products by rapidly producing foods with diverse designs and unique structures [6]. Severini et al. [7] used traditionally inedible insects to prepare nutritious protein–starch complexes for enriching wheat flour dough used in snack production. As the primary macronutrients in food materials, proteins and carbohydrates provide most of the energy required for human physiological activities. Therefore, food materials primarily composed of proteins and carbohydrates have become the main focus in 3D-printed food development. However, a single food material often cannot satisfy the requirements for successful 3D printing or meet modern consumer expectations regarding nutrition, color, shape, and taste [8]. Therefore, optimizing the types and proportions of raw materials can improve the structural properties of 3D printing materials, thereby enhancing their printability and shape fidelity. Liu et al. [9] ground glutinous rice, indica rice, and japonica rice into powder, then tuned mixture ratios by adjusting the water dosage. The extrusion performance of rice paste was characterized, and cooked molded specimens were analyzed by measuring in vitro starch digestibility as well as structural integrity for comprehensive quality evaluation. The results showed that, before cooking, the formulation with glutinous rice flour and water mixed at a 10:9 mass ratio achieved the best printing performance and the highest printing precision. After cooking, glutinous rice dough exhibited poor structural stability because of its high amylopectin content, whereas dough prepared from japonica and indica rice maintained good structural stability. Huang et al. [10] found that brown rice supplemented with a mixture of xanthan gum and guar gum exhibited the best printability and moderate hardness, with minimal deviations in the printed diameter and height. Microstructurally, the printed surface was smooth and free of obvious fractures. Vieira et al. [11] fabricated extrusion-manufactured functional cookies enriched with Arthrospira platensis. All cookie paste blends evaluated possessed favorable extrudability, generating uniform extruded strands whose diameters matched the nozzle bore size and retaining geometric conformity to the preset digital 3D model following deposition. Moreover, incorporating Arthrospira platensis yielded cookie specimens with distinctive visual aesthetics. Beyond raw material composition, manufacturing settings during additive fabrication also govern the outer contour and comprehensive quality of shaped food samples. Masbernat et al. [12] also developed a printable formulation composed of flour and water and applied specific thermomechanical treatments based on water content and starch gelatinization to achieve rheological and textural properties suitable for 3D printing.

Therefore, raw material characteristics (e.g., composition ratio and fluid viscosity), material printability (e.g., gel-forming capacity, rheological performance, melting temperature, and glass transition point), and manufacturing setup variables (e.g., printing nozzle height, diameter, and material flow rate) constitute the three core elements governing high-accuracy additive food fabrication [13]. In this study, wheat flour and citrus pectin powder were used as the primary raw materials to prepare 3D-printed dough, and the effects of the water, citrus pectin, and wheat flour ratios on printing quality were investigated. In addition, key manufacturing settings for food extrusion, including compression pressure, nozzle travel speed, needle bore size, and internal infill density, were characterized and fine-tuned to determine a set of parameters applicable to high-performance 3D food printing.

## 2. Materials and Methods

### 2.1. Materials

Commercial wheat flour (Jinshahe Flour Manufacturing Group Co., Ltd., Xingtai, China), citrus pectin powder, and water were bought from a local supermarket (Yonghui Superstores Co., Ltd., Chongqing, China) to prepare the food inks. Citrus pectin powder was purchased from DSM-Firmenich Pectin (Yantai) Co., Ltd. (Shandong, China).

### 2.2. Dough Preparation for Extrusion

The extrusion performance of the paste was explored by adjusting the proportions of wheat flour, citrus pectin powder, and water (Table 1). After the ingredients were mixed with water, each sample was kneaded with a dough mixer for ≥10 min until a uniform paste formed. Once blending finished, all prepared samples were preserved at 4 °C for follow-up testing.

### 2.3. 3D Printing Process

The 3D printing system comprised a feeder, extrusion system equipped with an air pump, and X–Y–Z positioning unit (Wiiboox Sweetin, Nanjing, China). The height of the printing nozzle was adjusted by modifying the feeder position on the printing equipment. In this study, all samples were printed at room temperature with a single nozzle. During loading of the food ink into the syringe barrel, the raw material was firmly compacted from the barrel’s top downwards to avoid trapped air bubbles, which could cause defects such as discontinuous extrusion during printing. Compressive pressure forced the material through a printing nozzle with a fixed inner diameter. The extruded material was then deposited and stacked onto a flat plastic film according to a printing path preset in the control program. The printing surroundings were kept at room temperature during all trials. The movement and positioning in the printing procedure were regulated via a self-designed JAVA computer program with a microcontroller. The relative positions along the X-, Y-, and Z-axes were controlled using computer-aided design, which guided the nozzle along a predefined printing path. A simple pumping unit relying on air pressure to squeeze the dough out of the syringe was adopted for the 3D food printing tests. The dough was extruded onto the printing platform by air pressure, which was tuned based on the rheological characteristics of the dough [14].

### 2.4. 3D Structure Analysis

The 3D-printed structures used in this experiment were a cone (D = 24 mm, H = 20 mm) and a cylinder (40 × 40 × 10 mm^3^). The dimensions of the printed cones and cylinders, including length, width, and height, were measured using a Vernier caliper. Each formulation was printed and measured in six replicates (*n* = 6). The photographs presented correspond to the samples that most closely represented the average experimental results. In short, photos of the printed models were captured mid-print and right after fabrication, and their forming performance was visually graded with a five-point scoring standard (1 = extremely poor; 5 = excellent). Forming accuracy and structural stability were considered in the print quality assessment.

### 2.5. 3D Printing Printability

3D models for two objects, a cone (filled in, designed height = 20 mm, diameter = 24.0 mm) and a cylinder (hollow, designed stacking layers = 8, diameter = 40.0 mm), were generated using Slic3r software (version 4.1.1). The printing parameters were as follows: printing pressure = 400 kPa, nozzle diameter = 1.0 mm, and printing speed = 20 mm/s. After printing, the structures were photographed immediately from front and top views after 30 min to assess shape retention and stability. The 3D-printed cubes and cylinder were measured immediately after printing and after storage at room temperature (25 °C) for 30 min. The diameter was measured using ImageJ software (version 1.52a, NIH, Washington, DC, USA). Six independent cubes were printed for each formulation. Printed figure results were compared with the designed model to see deviations [15]. Percentage errors were then calculated for each dimension using Equation (1):

(1) Perror = (X measured − Y designed)/Y designed × 100

where Perror represents the percentage error, X measured is the average length (width or height), and Y designed is the corresponding designed length derived from the CAD design.

### 2.6. Dough Structure Characterization

The texture profile of the 3D-printed products was analyzed following the method proposed by Liu et al. [16]. The effects of material composition and compression pressure during 3D printing on the structural characteristics were investigated using sample B before printing and sample B printed at 350 kPa. After sample preparation, all test pieces were placed on the test platform and subjected to texture profile analysis (TPA) with a P/0.5 probe. The moving speed of the probe was fixed at 2 mm/s before, during, and after detection. Every specimen was compressed to 50% of its initial height; the test parameters included a 5 s compression gap, 5 g trigger force, and 200 pps sampling rate. Hardness, adhesiveness, springiness, and gumminess were recorded throughout detection, and the mean values from six measurements (*n* = 6) were used for analysis.

### 2.7. Statistical Analysis

All data were expressed as mean ± standard deviation (SD). All results were presented as the mean ± standard deviation. All analyses were conducted in six replicates (*n* = 6). Statistical analysis was performed using one-way analysis of variance followed by Duncan’s test using SPSS software (SPSS version 12.0, SPSS Inc., USA). Duncan’s multi-range test was used to compare the differences among treatments. Differences between means were considered significant at *p* < 0.05.

## 3. Results and Discussion

### 3.1. Citrus Pectin Dough Printability

The formulations of the different dough samples are presented in Table 1. The samples were printed as a cone (D = 24 mm, H = 20 mm, filling ratio = 30%) and a cylinder (D = 40 mm, stacking layers = 8, filling ratio = 30%) using a nozzle with a diameter of 1 mm, a printing speed of 20 mm/s, and a compression pressure of 350 kPa. Printability refers to the ability of a food material to maintain its intended shape during and after extrusion under the control of a 3D printer, thereby enabling the successful fabrication of food products with diverse geometries and good structural stability. All eight dough formulations were successfully printed into conical and cylindrical structures under a compression pressure of 350 kPa, a printing speed of 20 mm/s, and an internal filling ratio of 30%. The shapes and print quality of the cone- and cylinder-shaped samples after completion of the printing process are shown in Table 2.

At a fixed water content, the amount of citrus pectin added to the wheat flour was varied to investigate the effect of pectin concentration on the printability of the dough. In the printing stage, it was observed that the amount of pectin added affected printing performance within the experimental concentration range. As shown in Table 2, in the absence of pectin, the printing process became progressively more difficult as the number of printed layers increased, and the frequency of structural deformation also increased. As the pectin concentration increased, the volume of the printed samples varied considerably. This effect may be attributed to the ability of hydrocolloids to strengthen the interactions between gluten and starch granules, thereby forming a stronger three-dimensional network structure and improving the rheological properties and printability of the dough [17]. Pectin was distributed throughout the continuous phase of the wheat flour dough and therefore contributed to forming a dense dough structure. Within an appropriate concentration range, increasing the pectin content enhanced the tensile strength and cohesive strength of the dough, resulting in a compact and elastic dough structure.

Applying pectin in bread making can effectively increase dough volume and improve freshness, stability, and softness. This effect may be attributed to the redistribution of water molecules among starch, gluten, and other components (such as added pectin), which influences water mobility and gluten network development [18,19]. Within an appropriate concentration range, dough stability time increased as the pectin concentration increased, indicating greater resistance to shear during the forming process [20]. This finding suggests that the disulfide bonds within the gluten protein network remained firmly bound and were less susceptible to disruption [21]. Beyond this concentration range, pectin may interact with gluten through electrostatic interactions, resulting in repulsion between adjacent gluten chains, reduced cross-linking, and a porous dough microstructure. Consequently, the gluten network may partially disintegrate, leading to reduced dough stability. Correa et al. [22] reported similar findings, demonstrating that anionic hydrocolloids (λ-CGN, carboxymethylcellulose, and pectin) could bind with gluten to form hydrophilic complexes via electrostatic interactions, thereby inducing conformational changes in gluten. Excess pectin may not be uniformly incorporated into the flour matrix, reducing the amount of pectin that effectively participates in network formation [23]. This may explain why samples C and G exhibited similar printing performance. In sample G, the excess pectin may have partially disrupted the gluten network, resulting in slightly blurred boundaries.

The dimensions of the printed conical and cylindrical structures can be intuitively evaluated by measuring their diameters. Among the conical samples, sample B had the largest diameter (34.3 ± 0.5 mm), whereas sample E had the smallest diameter (20.3 ± 0.3 mm). Among the cylindrical samples, sample D exhibited the largest diameter (54.7 ± 1.0 mm), whereas sample G had the smallest diameter (46.0 ± 1.5 mm). Based on the printability evaluation of samples A–H, samples C, F, G, and H exhibited better printing performance than the other samples for both the conical and cylindrical structures. In particular, observations of the cylindrical samples showed that the internal filling patterns of samples C and G exhibited better alignment than those of samples F and H. Similarly, observations of the conical samples showed that although samples C, F, G, and H all maintained their conical shapes, the volumes of samples C and G were more appropriate, whereas sample F exhibited an excessively small volume and sample H exhibited an excessively large volume. This difference may be attributed to discontinuous material extrusion in sample F during printing, whereas partial collapse of the internal structure may have occurred in sample H, resulting in line displacement and an increased apparent volume after layer stacking.

In contrast, the cones produced from samples A, D, and E exhibited twisting and deformation during the stacking process. Observations of the cylindrical samples showed that the internal filling patterns of samples A and E were more irregular than those of sample D. Sample D exhibited blurred edges, which may have resulted from progressive dough deformation as the printing height increased. Sample A exhibited more discontinuous extruded filaments than sample E and therefore showed a higher frequency of filament breakage, possibly because the addition of pectin increased the water-holding capacity of the dough. This effect may be attributed to the ability of pectin to reduce the pore size of the wheat flour dough while increasing the number of smaller pores, thereby forming a dense and uniform network structure [24]. This structural modification enhanced water binding, resulting in improved water-holding capacity [25]. The increased volume observed in the middle region of sample B may also have been caused by blurred boundaries between adjacent internal layers, resulting in unstable layer stacking and structural deformation. Sample H exhibited a relatively uniform overall volume, although slight deformation was observed at the top because of layer superposition. Overall, sample E exhibited incomplete and discontinuous internal filling patterns together with pronounced structural deformation, resulting in the lowest overall score (3.1 ± 0.2). In contrast, samples C and G exhibited the best printing performance, with overall scores of 4.6 ± 0.2 and 4.5 ± 0.3, respectively.

### 3.2. Effect of Compressive Pressure on Citrus Pectin Dough Printing Quality

To evaluate the printability evaluation of samples prepared with different wheat flour, citrus pectin, and water formulations, we used set parameters for printing (cone: H = 20 mm, D = 24 mm, filling ratio = 30%; cylinder: D = 40 mm, filling ratio = 30%, stacking layers = 8; printing parameters: printing pressure = 400 kPa, nozzle diameter = 1.0 mm, printing speed = 20 mm/s). The success of 3D food printing relies on both the inherent characteristics of raw materials and extrusion process parameters, which directly affect the quality of the final product. Because samples C and G exhibited similar printing performance (Table 2), samples A, C, F, and H were selected for further comparison to investigate the effect of compressive pressure on print quality. The molding performance of these specimens was assessed under various extrusion pressure levels (Figure 1a). The test adopted a 1 mm nozzle bore size, 30% internal infill density, and 20 mm/s travel speed, with a mooncake-forming model (D = 30 mm, Figure 1b), so as to identify the optimal extrusion pressure. Figure 1c presents the molding score data of the four test samples under extrusion pressures ranging between 250 and 450 kPa at an increment of 50 kPa. The highest print quality for samples C and H was achieved at a compressive pressure of 350 kPa. Among all samples, sample C achieved the highest score (4.45 ± 0.18) under the 350 kPa printing condition, indicating the best printing performance. The dough printed under this condition exhibited clear outlines, well-defined edges, good layer stacking, and continuous filaments, which was consistent with the corresponding photographs. At compressive pressures of 400 and 450 kPa, both samples A and F exhibited severe shape distortion or even completely lost their original shapes, indicating that excessive pressure caused over-extrusion of the dough, leading to collapse of the deposited filaments. Consequently, the printed structures exhibited poor overall stability and severe deformation. This behavior may be explained as follows: if the extrusion pressure was excessively low, the applied force was insufficient to extrude the material uniformly through the nozzle. Consequently, irregular printing patterns were generated (Figure 1a), including reduced printing area and irregular filament arrangement. Conversely, when the compressive pressure was too high, the material was extruded too rapidly from the nozzle, causing filament bending and unstable layer stacking [26]. As a result, samples with weaker structural integrity exhibited greater deformation and less distinct filament outlines [27].

### 3.3. Needle Speed

Printing speed refers to the rate at which the printing nozzle moves along the X-, Y-, and Z-axes during the printing process. A high printing speed reduces the time required to fabricate an object but may also affect printing accuracy. Based on the preliminary experimental results, sample C was selected to investigate the effect of printing speed on print quality and to determine the optimum printing speed for 3D printing. During this experiment, the following parameters were fixed: nozzle diameter, 1 mm; compression pressure, 350 kPa; and internal filling ratio, 30%. When the printing speed was too low, material extrusion lagged behind the nozzle movement and could not accurately follow the predefined printing path, resulting in filament bending, overlapping, and localized thickening. After the material had been deposited, the previously printed layers could also be scratched or deformed because of an inappropriate nozzle height during subsequent printing. Conversely, when the printing speed was too high, the extruded material could be stretched and dragged, producing discontinuous filaments and a nonuniform printed structure. As shown in Figure 2a–e, when the nozzle moved at a speed of 10 mm/s, the filament outlines began to overlap and became blurred, while the internal voids became smaller. When the nozzle movement speed was too high (25 or 30 mm/s), the material extrusion rate did not match the printing speed. As a result, the deposited filaments became bent and deformed, and the internal voids became irregular. When the printing speed was 15 mm/s, the printed samples exhibited the highest print quality (Figure 2b) (4.52 ± 0.14), with uniform filament thickness, clear outlines, and excellent layer stacking. Yang et al. [28] found that a printing speed of 20 mm/s produced the most irregular printed structure, which may have been related to the dough viscosity. Furthermore, when the printing speed was too high (30 mm/s), the corners of the printed product could not be completely formed, and the extruded filaments were prone to breakage because of dragging, resulting in a large deviation between the printed product and the designed model. Similarly, Dong et al. [29] reported that excessively high printing speeds caused filament dragging or breakage, whereas excessively low printing speeds resulted in unstable material flow and blurred filament patterns. Longitudinal cross-sectional observations and microstructural analysis showed that some deposited filaments were stacked either too high or too low, resulting in nonuniform spacing within the printed structures. Accordingly, 3D dough printing differs from conventional fused deposition modeling (FDM) plastic 3D printing. The needle movement speed and printing effect are significantly negatively correlated. If the needle moves too fast, it will result in insufficient extrusion, poor interlayer bonding, structural collapse, and a rough surface. Possible reasons include the high viscosity of the dough, which makes the extrusion unable to keep up with the moving speed, causing discontinuous material accumulation. If the needle moves too slowly, excessive material accumulation, deformation, prolonged printing time, and nozzle clogging occur. The reason is that when the output volume per unit time remains constant, moving at a slow speed will cause the material to accumulate excessively. Only when the extrusion speed matches the moving speed can the food-grade material be uniformly deposited [30].

### 3.4. Stackable Height of Citrus Pectin Dough

On the basis of the foregoing data, the optimal extrusion pressure of 350 kPa together with a constant travel velocity of 15 mm/s was adopted to assess the maximum stackable height of sample C (Figure 3). The maximum stackable height of the food samples depends not only on the material properties but also on the printing speed and material flow rate [31]. After 3D printing, all tested samples maintained the cylindrical shape of the mooncake; however, the lower portion was wider than the upper portion. This deformation may be attributed to compression of the lower layers by the weight of the upper dough layers during printing. As the filling ratio increased, the deformation of the bottom region became pronounced, which may be because a high filling ratio increased the weight of the printed structure at the same height. The degree of bending also differed among samples with filling ratios of 10%, 30%, 50%, 70%, and 100%. At a filling ratio of 10%, severe edge deformation occurred when the printing height reached 35.3 ± 3.1 mm (Figure 3f). If the printing height continued to increase, dough structure collapse was expected. Therefore, 35.3 ± 3.1 mm was considered the maximum achievable printing height under this condition. Pronounced deformation and severe irregularities in filament stacking can also be observed in the printed samples (Figure 3a). At a filling ratio of 30%, when the printing height reached 36.2 ± 3.2 mm, the internal filling pattern at the top cross-section of the sample became irregular, the outlines became blurred (Figure 3b,f), and structural deformation occurred earlier than that observed at a filling ratio of 10%. When the filling ratio was increased to 50%, the maximum printing height reached 38.0 ± 2.3 mm, with bending deformation and partial internal collapse observed at the top cross-section (Figure 3c,f). At filling ratios of 70% and 100%, notable truncation was observed. However, because the printed samples exhibited a narrow top and wide bottom, the center of gravity remained relatively low, allowing greater printing heights to be achieved. Nevertheless, severe deformation occurred, and the overall printing performance was inferior to that obtained at a filling ratio of 50% (Figure 3d–f). Overall, improved printable heights generally indicated better dimensional stability of the printed structures [32]. Notably, deposited filament deformation consistently occurred at the top of the cylindrical samples, and top deformation was more pronounced than that at the bottom (Figure 3a–e). This phenomenon may be attributed to the increasing compressive load exerted on the lower layers as the printing height increased, while the upper layers remained insufficiently supported because of the flexibility of the dough. Consequently, although the lower portion of the printed sample had a larger cross-sectional area than the upper portion, deformation of the newly deposited filaments was more pronounced near the top of the structure than near the bottom. 3D printing dough usually uses flour as the printing material, and its properties are different from those of plastic filament. The filling rate and layer height have a mutually restrictive relationship; the larger the layer height, the higher the required filling rate to maintain structural stability. The dough paste-like substance makes it difficult to support the combination of a large layer height and low filling. High filling is suitable for load-bearing structures, but the dough is prone to deformation due to excessive accumulation, and the surface quality deteriorates. Only an appropriate filling rate is suitable for mass production, but the adhesion of the material needs to be ensured [33].

### 3.5. Effect of Internal Filling Ratio on the Printing Height and Printing Quality

The internal filling ratio has a substantial influence on the appearance and structural quality of the printed product. A low filling ratio decreases the time required for printing, whereas a high filling ratio increases the mechanical strength of the printed structure but also requires additional raw material. Sample C was printed using a mooncake model (D = 30 mm, stacking layers = 8) with filling ratios of 10%, 30%, 50%, 70%, and 100% (Figure 4a). The effect of the internal filling ratio on the print quality of sample C was evaluated under a fixed nozzle diameter of 1 mm, a compressive pressure of 350 kPa, and a printing speed of 15 mm/s. The results showed successful fabrication of the 3D-printed samples at filling ratios of 10%, 30%, and 50% (Figure 4b). However, as the printing height increased, the extent of deformation varied among the different filling ratios. A low filling ratio (10%) did not provide sufficient structural support to maintain the printed shape, whereas high filling ratios (70% and 100%) promoted dough accumulation within the printed structure, thereby reducing printing accuracy. In addition, the greater mass of the upper layers increased compressive loading on the lower layers, limiting the achievable printing height. At a filling ratio of 30%, the overall appearance and structure of the printed samples, as well as the number and size of the internal voids, closely matched the designed structure. This may be attributed to the fact that an appropriate filling ratio provided sufficient structural support to maintain the geometry of the printed product without excessive material accumulation within the internal structure, thereby resulting in good printing accuracy. However, the printing height under this condition was not the highest, which differs slightly from the findings of Liu et al. [14]. They achieved the greatest printing height together with the highest printing accuracy, which may be attributed to variations in the properties of the raw materials used. At a filling ratio of 50%, the maximum printing height was achieved (9.6 ± 0.2 mm); however, the internal voids were not uniformly distributed. In addition, deformation caused by extrusion of excess material was observed in the printed samples because of the continuous deposition of the dough during printing. When the filling ratios were increased to 70% and 100%, the internal filament patterns became almost indistinguishable, and the bottom of the printed structures expanded. This may be because the high filling ratio reduced the spacing between adjacent extruded filaments, while the increasing weight of the upper layers increased the compressive load on the lower layers as the printing height increased, thereby causing deformation at the base of the printed samples. In addition, this deformation may also have resulted from layer misalignment caused by the intersecting printing paths within the infill region, which is consistent with the findings of Chen et al. [34].

### 3.6. Needle Diameter

From the foregoing analytical results, sample C was chosen for further testing with extrusion pressure maintained at 350 kPa, internal infill density set to 30%, and a constant travel velocity of 15 mm/s. As displayed in Figure 5, no positive correlation was observed between print quality and nozzle diameter. When a 0.25 mm nozzle diameter was used, the applied pressure was insufficient to extrude the dough smoothly through the nozzle. Consequently, the material accumulated at the nozzle tip, and only a small amount of dough was extruded (Figure 5a), resulting in the poorest printing performance. This may be because a small nozzle diameter improves the smoothness and dimensional accuracy of the printed product but also increases the flow resistance during extrusion, making it more difficult for the dough to pass through the nozzle. As shown in Figure 5f, the best printing performance was achieved with a nozzle diameter of 1 mm. Under this condition, the printed samples exhibited uniform geometry and well-defined internal filling patterns, maintained their structural integrity over time, and achieved the highest print quality score (4.39 ± 0.20). When the nozzle diameter was 0.5 mm (Figure 5b), the internal filling pattern of the sample remained relatively regular. However, numerous filament discontinuities were observed, and the thickness of some deposited filaments was nonuniform. This may be because the nozzle diameter was too small, resulting in nonuniform stress distribution within the dough and an unstable extrusion rate. Nozzle bore size exerts a direct influence on the dimensional precision and surface texture of printed specimens. A small nozzle diameter generally improves the dimensional accuracy and surface finish of the printed structure but also increases the printing time. Under constant air pressure, a small nozzle diameter subjects the dough to greater extrusion resistance, making it more difficult to preserve the integrity of the internal structure during extrusion. In contrast, larger nozzle diameters produce relatively rougher printed surfaces and poorer print quality. This phenomenon arises since a greater quantity of feedstock extruded through the nozzle diminishes dimensional precision, which makes the printed architecture drift away from the target design geometry. Liu et al. [35]. studied the effects of nozzle diameter (1.0, 1.5, and 2.5 mm) on the printing characteristics of gelatin, corn starch, and egg white protein mixtures and reported similar findings. As the nozzle diameter increased, the extrusion pressure gradually decreased, whereas the dimensional resolution and surface quality of the printed products deteriorated.

### 3.7. TPA of Citrus Pectin Dough

The hardness, springiness, adhesiveness, and gumminess of the dough not only have important influences on dough processing during production but also reflect the softness, internal structure, and texture of the dough products. Therefore, these parameters can be used to evaluate dough quality. In food TPA, hardness refers to the maximum force required to compress the sample during the first compression cycle, corresponding to the first peak force recorded by the texture analyzer. This parameter reflects the structural strength of the sample during compression. Within a certain range, lower hardness indicates a softer dough, greater springiness indicates better recovery after deformation, and greater cohesiveness reflects stronger internal bonding and improved dough handling properties [36]. Figure 6 shows the TPA results for samples A, C, F, H, and AP (after printing). All reported values represent the mean results obtained from six independent measurements (*n* = 6). Among all samples, sample A exhibited the highest hardness (158.06 ± 24.78 g). After pectin was added, the hardness decreased by 53% compared with that of sample A.

The addition of pectin effectively reduced the hardness of the dough (Figure 6a), likely because the presence of this polysaccharide affected gluten–starch network formation, thereby reducing dough firmness. Consequently, the dough became soft. Furthermore, as the pectin content increased, the hardness of the samples gradually decreased. This may be attributed to the low flour content and high polysaccharide content in the formulation, which promoted hydrogen bonding between water molecules and polysaccharides, thereby reducing water availability for gluten hydration and swelling. Dough hydration occurs in two stages. Initially, when water absorption is limited, a continuous gluten network cannot be formed. Subsequently, as water diffuses into the protein matrix, the gluten proteins absorb sufficient water to develop a continuous gluten network. Therefore, under the same mixing conditions, the dough containing pectin exhibited a weaker gluten network than the dough without pectin (*p* < 0.05). The hardness of the sample AP (65.94 ± 12.89 g) was significantly lower than that of sample C, indicating that the dough structure became weaker after printing and that the printed samples were softer than the unprinted dough. Innun et al. [37] reported that globin glycated with sugars (psicose, fructose, and glucose) through the Maillard reaction reduced dough firmness by approximately 30% compared with nonglycated globin during breadmaking. They suggested that the presence of low-molecular-weight sugars increased the osmotic pressure outside the protein molecules, thereby reducing water diffusion into the proteins and limiting gluten development, ultimately decreasing dough hardness. Furthermore, this reduction may be attributed to the layer-by-layer deposition process used in 3D printing. During printing, the dough was extruded and deposited sequentially to form the final structure. Consequently, adjacent layers were primarily joined through physical contact rather than complete fusion, resulting in weaker interlayer bonding [38]. Adhesiveness describes the energy needed to break the intermolecular attraction between food surfaces and contacting substances such as the tongue, teeth, and palate. As the pectin content increased, the interactions among gluten proteins were weakened, increasing the tendency of the dough to adhere to the probe during TPA, and the adhesiveness increased significantly (*p* < 0.05). After printing (sample AP), the adhesiveness of the dough also tended to increase. This may be related to the higher moisture content of the dough after extrusion, which was observed during printing. Under the applied extrusion pressure, moisture migrated from the upper portion of the dough to the lower portion that was about to be extruded. Furthermore, the increased water-holding capacity of the printed dough may have enhanced interactions among the dough components, thereby contributing to the increased adhesiveness. When the pectin content was 0.7 g (sample H), the dough exhibited the highest adhesiveness. Hydrophilic substances, such as pectin, can increase dough adhesiveness because of their hydrophilic functional groups [39]. Springiness refers to the ability of deformed dough to recover its original shape after the removal of an applied force and reflects the elasticity of the dough, which is an important textural attribute influencing consumer acceptance. Figure 6c shows the springiness of the dough. When the pectin content was 0.7 g (sample H), the dough exhibited the lowest hardness before printing (74.30 ± 7.23 g) and the highest springiness (0.91 ± 0.03), indicating that the dough was more readily extruded through the nozzle. Comparison of samples C and AP showed that the springiness increased after printing, suggesting that the printing process improved the textural properties of the dough. This may be because the pressure applied during printing altered the interactions among the dough components, thereby affecting the structural characteristics of the dough and its texture. After pectin addition, the gumminess increased significantly, with values for samples C, F, and H being 40.56%, 41.70%, and 46.70% higher than that of sample A, respectively (Figure 6d). These results indicate that the addition of pectin and the application of 3D printing technology altered the internal structure of the dough, thereby improving the textural properties of the dough products to a certain extent. Furthermore, these findings suggest that pectin can serve as a modifier to alter the internal structure of dough and that the application of 3D printing technology has the potential to further enhance the textural quality of dough products.

## 4. Conclusions

In this study, 3D-printable dough was prepared using wheat flour and citrus pectin. All formulations achieved successful printing, with the best print quality obtained with pectin additions of 0.2 g, 0.5 g, and 0.7 g. To obtain superior forming quality, the optimal set of printing process parameters was determined as follows: 350 kPa extrusion pressure, 15 mm/s extrusion speed, 1 mm nozzle diameter, and 30% internal infill proportion. TPA results indicated that pectin can serve as a modifier to alter the internal structure of the dough, and that 3D printing improved its textural properties to a certain extent. Successful 3D-printed dough product fabrication demonstrated several advantages, including well-defined geometries and clear internal structural features. Therefore, incorporating pectin into the dough, along with the application of 3D printing technology, not only improved the internal structure of the dough but also expanded the design flexibility of dough-based products. Furthermore, this study provides useful information regarding the relationships between printable height, shape stability, and material properties of food inks for extrusion-based 3D food printing.

## Figures and Tables

**Figure 1:**
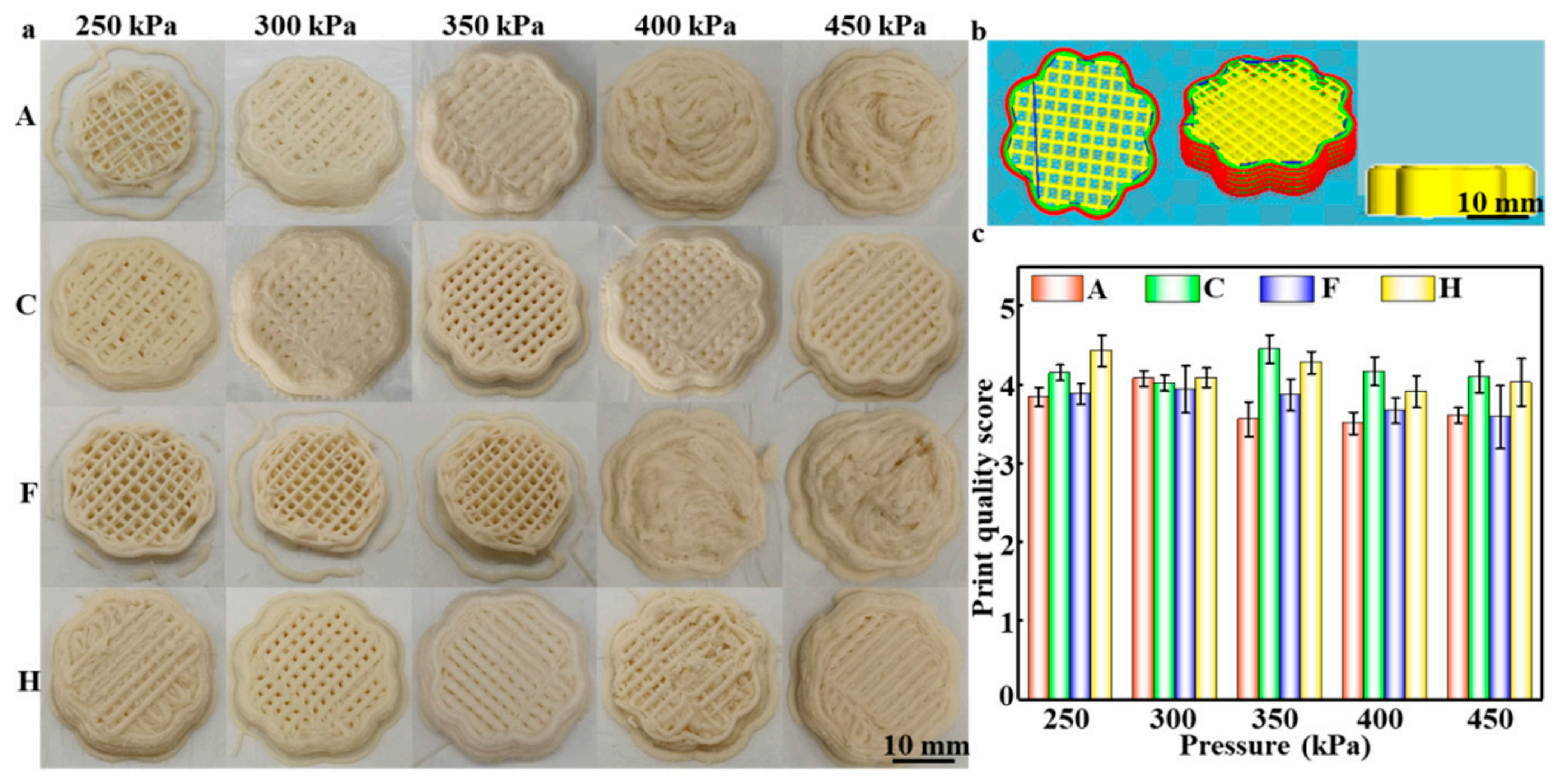
(**a**) Representative photographs of dough samples A, C, F, H printed under various compressive pressures (nozzle diameter = 1 mm, filling ratio = 30%, printing speed = 20 mm/s). (**b**) Schematic of the mooncake model (D = 30 mm) designed using the Slic3r software. (**c**) Influence of extrusion pressure on molding performance. Other printing parameters: printing speed = 20 mm/s and nozzle diameter = 1 mm.

**Figure 2:**
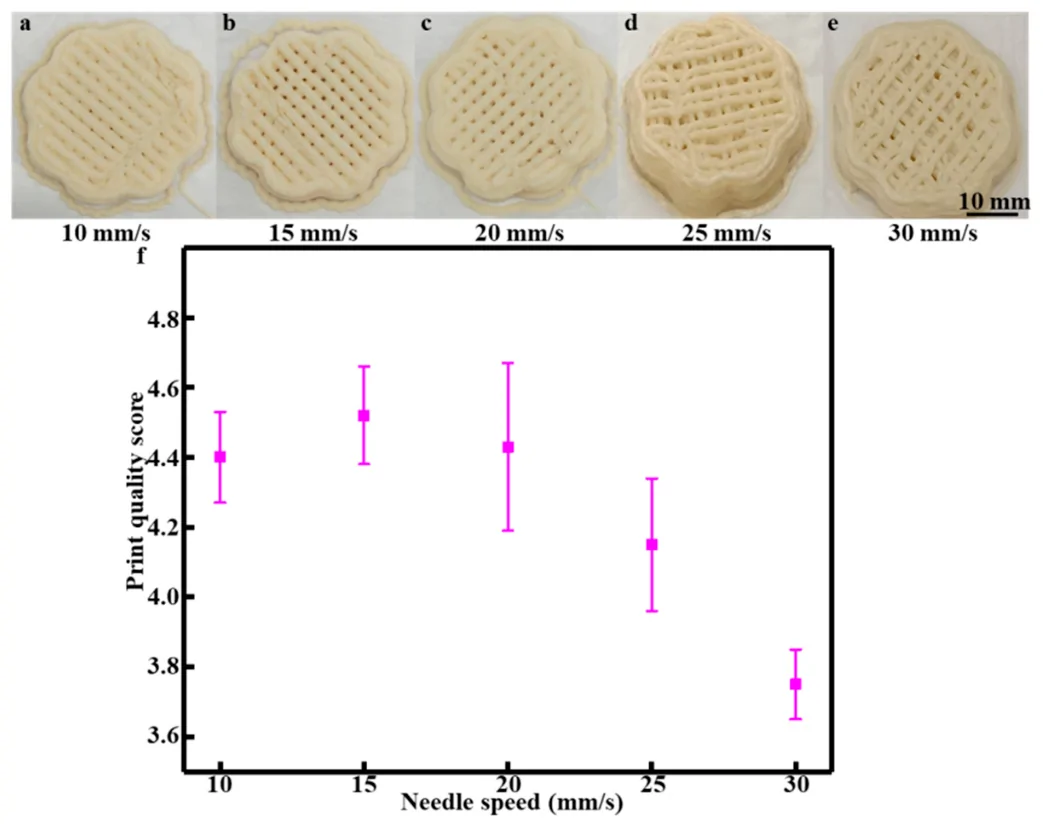
Print quality of dough sample C at printing speeds of (**a**) 10 mm/s, (**b**) 15 mm/s, (**c**) 20 mm/s, (**d**) 25 mm/s, and (**e**) 30 mm/s. (**f**) Molding performance scores under varied extrusion velocities. Other printing parameters: filling ratio = 30%, printing pressure = 350 kPa, and nozzle diameter = 1 mm.

**Figure 3:**
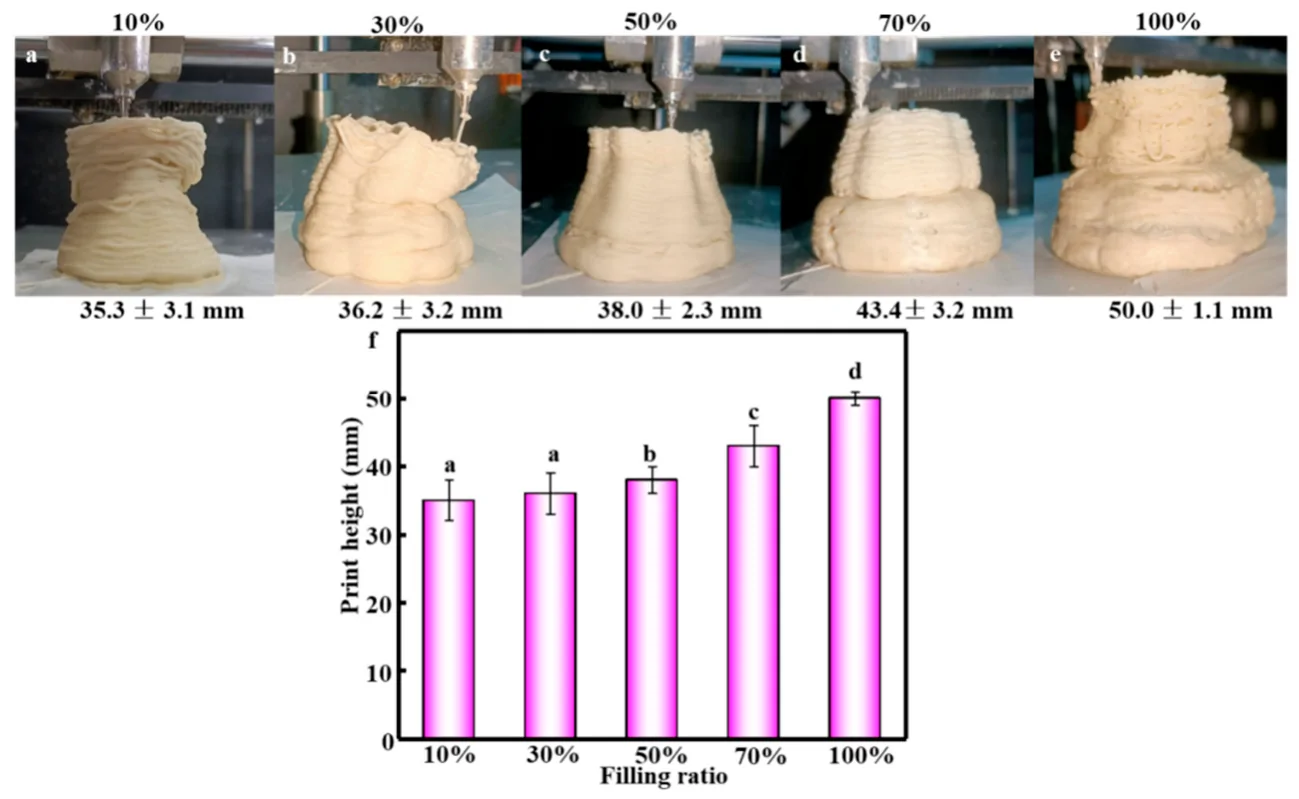
Geometries of 3D-printed dough samples (sample C) at different filling ratios: (**a**) 10%, (**b**) 30%, (**c**) 50%, (**d**) 70%, and (**e**) 100%. (**f**) Maximum printable heights at different filling ratios. Other printing parameters: printing speed = 15 mm/s, printing pressure = 350 kPa, and nozzle diameter = 1 mm. Different letters (a–d) indicate statistically significant differences (*p* < 0.05).

**Figure 4:**
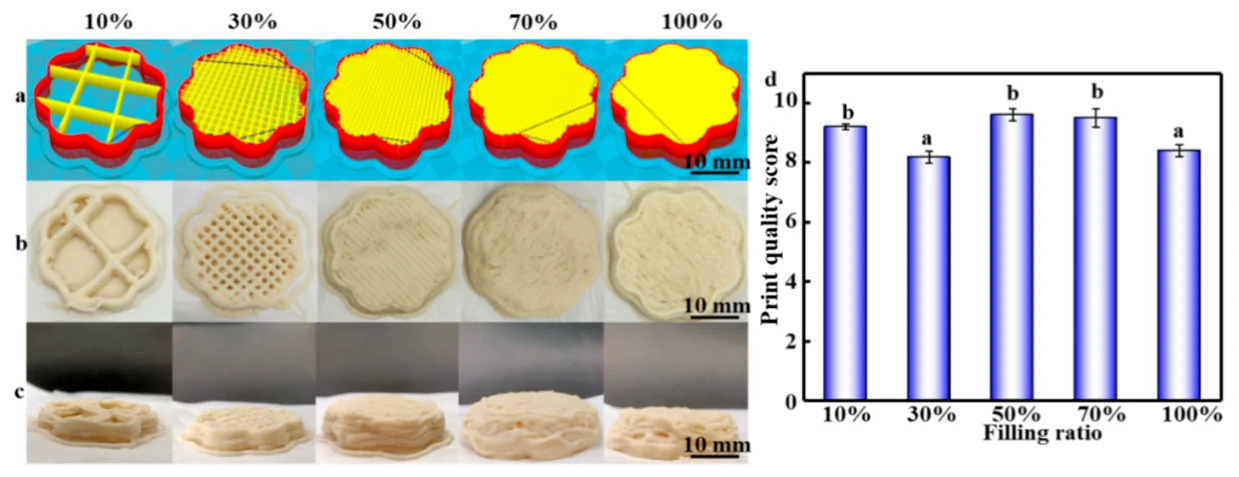
(**a**) Three-dimensional design of a cylindrical model with filling ratios of 10%, 30%, 50%, 70%, and 100%. (**b**) Representative photographs of dough fabricated at varied infill densities (top view). (**c**) Representative side-view photographs demonstrating the molding performance of printed dough with varied infill densities. (**d**) Print quality scores of sample C at different filling ratios. Other printing parameters: printing pressure = 350 kPa, nozzle diameter = 1 mm, and printing speed = 15 mm/s. Different letters (a,b) indicate statistically significant differences (*p* < 0.05).

**Figure 5:**
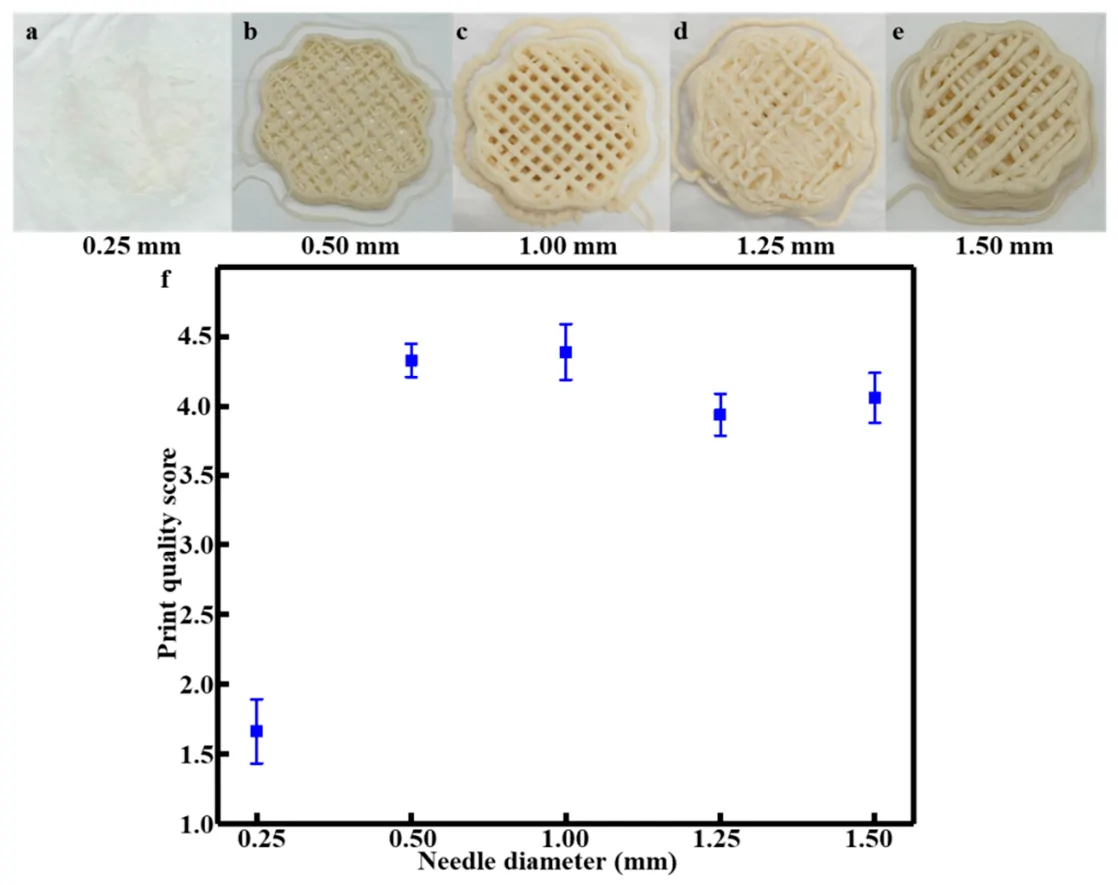
Representative photographs of dough sample C printed using nozzle diameters of (**a**) 0.25 mm, (**b**) 0.50 mm, (**c**) 1.00 mm, (**d**) 1.25 mm, and (**e**) 1.50 mm. (**f**) Print quality scores of sample C at different nozzle diameters. Other printing parameters: printing speed = 15 mm/s and printing pressure = 350 kPa.

**Figure 6:**
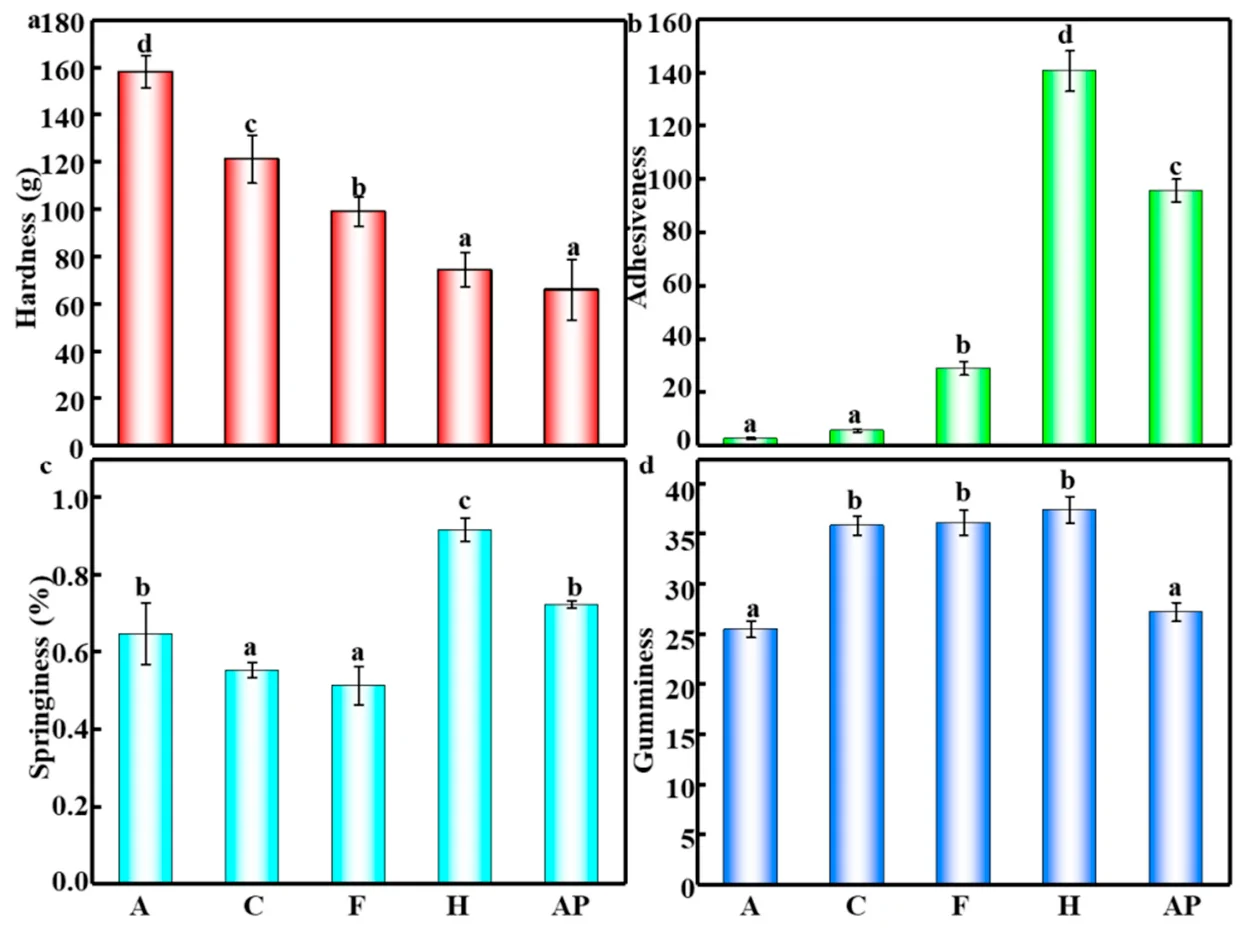
Texture profile analysis (TPA) parameters of different dough (samples A, C, F, H, and AP after printing at 25 °C): (**a**) hardness, (**b**) adhesiveness, (**c**) springiness, and (**d**) gumminess. Differences were considered statistically significant at *p* < 0.05. Different letters (a–d) indicate statistically significant differences (*p* < 0.05).

**Table 1:** Formulations of the samples prepared in this study.

Sample	Wheat Flour (g)	Citrus Pectin (g)	Water (g)	Total Mass (g)
A	50.0 ± 0.1	0 ± 0.1	35.1 ± 0.1	85.1 ± 0.1
B	48.1 ± 0.1	2.0 ± 0.1	35.0 ± 0.1	85.1 ± 0.1
C	46.1 ± 0.1	4.0 ± 0.1	35.0 ± 0.1	85.1 ± 0.1
D	44.0 ± 0.2	6.1 ± 0.1	35.0 ± 0.1	85.1 ± 0.1
E	42.1 ± 0.1	8.1 ± 0.1	35.1 ± 0.1	85.2 ± 0.1
F	40.1 ± 0.1	10.1 ± 0.1	35.1 ± 0.2	85.3 ± 0.2
G	38.1 ± 0.1	12.1 ± 0.1	35.0 ± 0.1	85.2 ± 0.1
H	36.0 ± 0.1	14.1 ± 0.2	35.1 ± 0.1	85.2 ± 0.1

**Table 2:** Representative photographs and printability evaluation of samples prepared with different wheat flour, citrus pectin, and water formulations (*n* = 6).

Sample	Cone	Diameter (mm)	Cylinder	Diameter (mm)	Print Quality	Observation
Model design	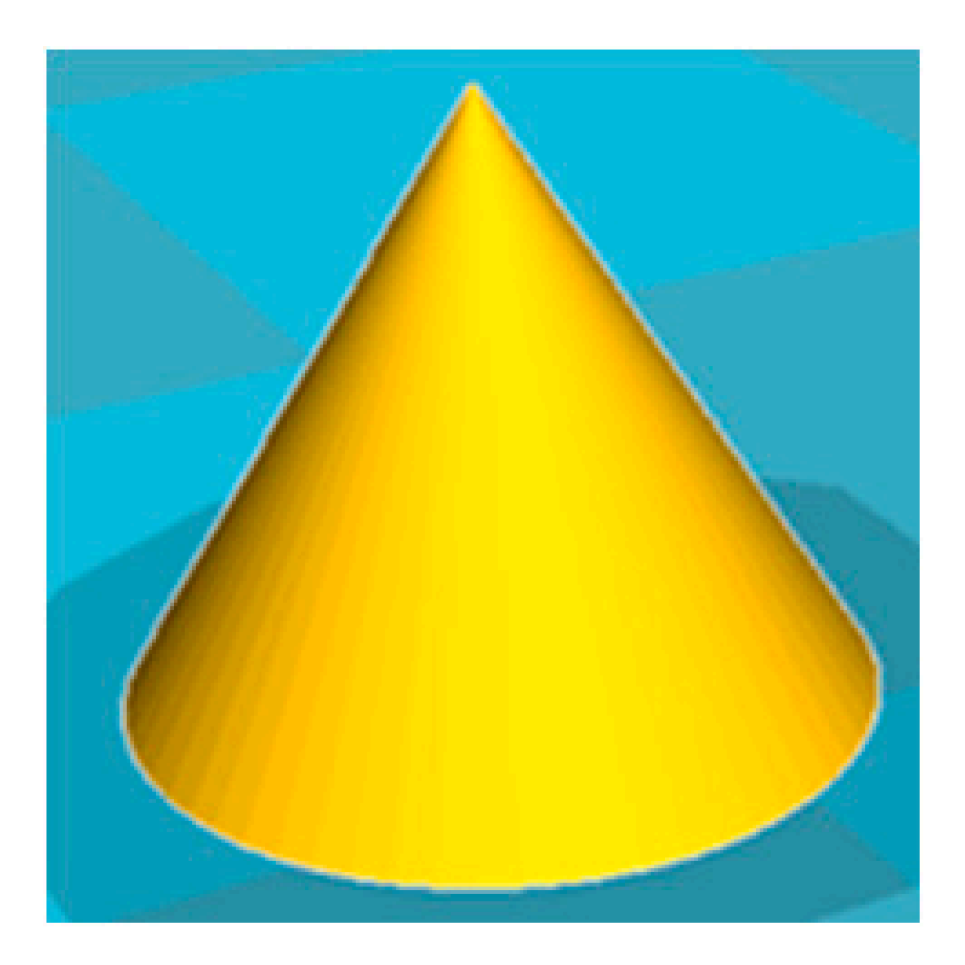	24.0	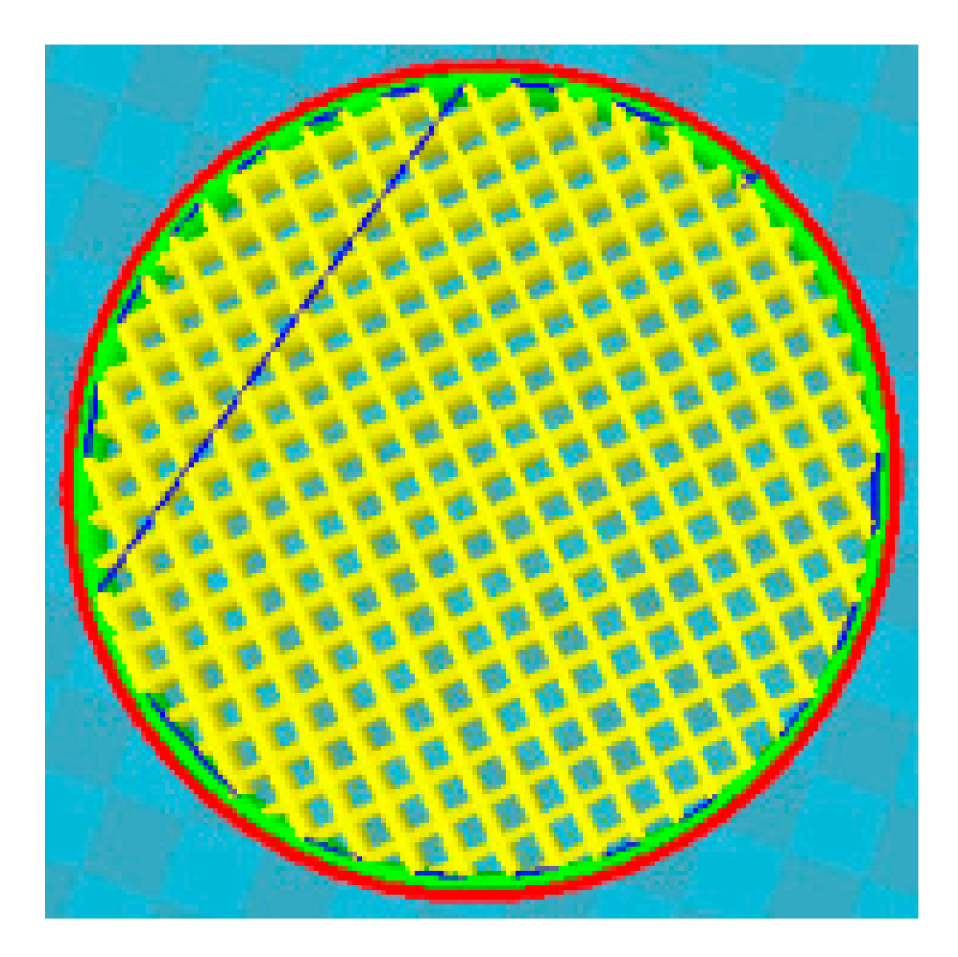	40.0	/	/
A	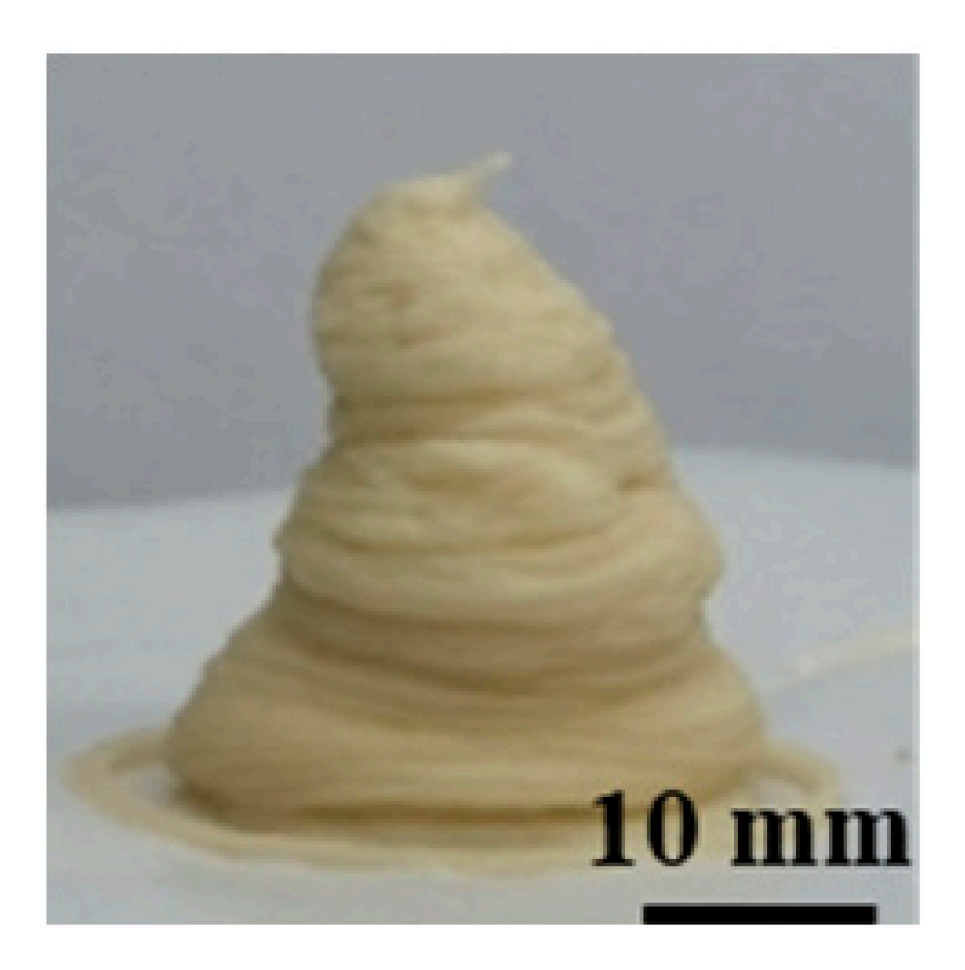	24.5 ± 0.2	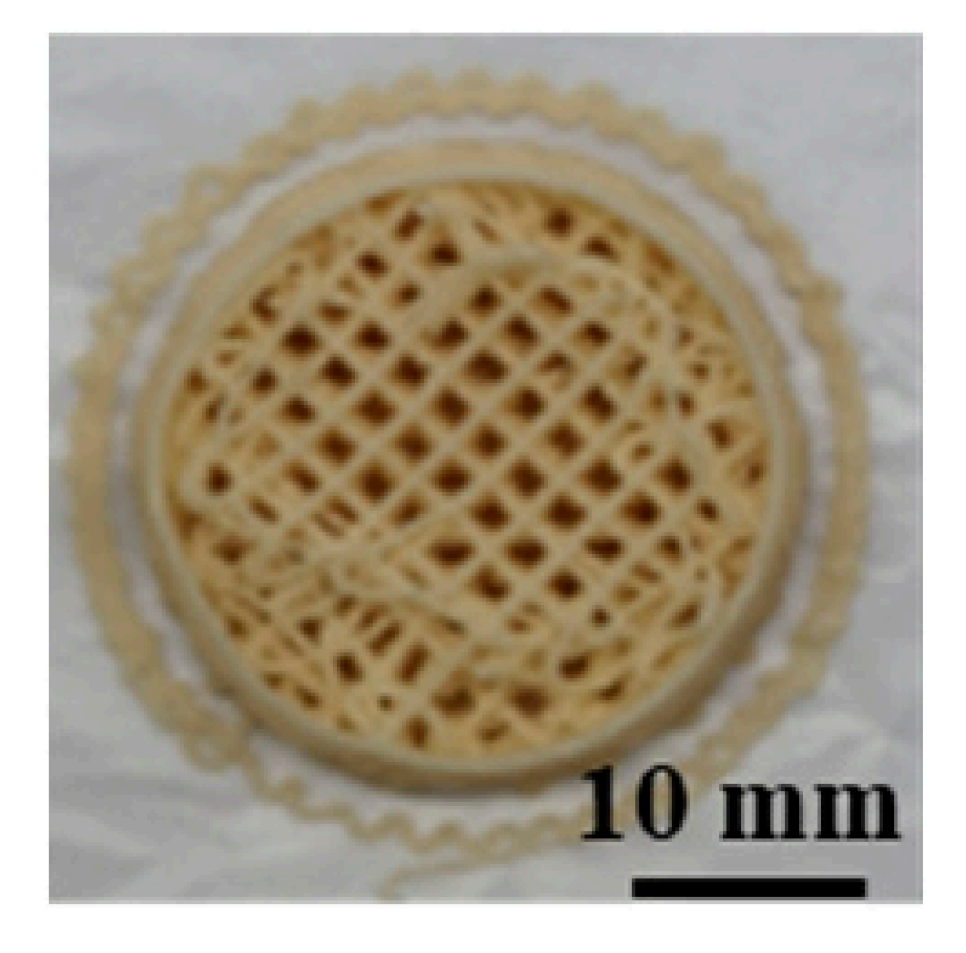	54.2 ± 2.1	3.6 ± 0.1	Irregular filament arrangement
B	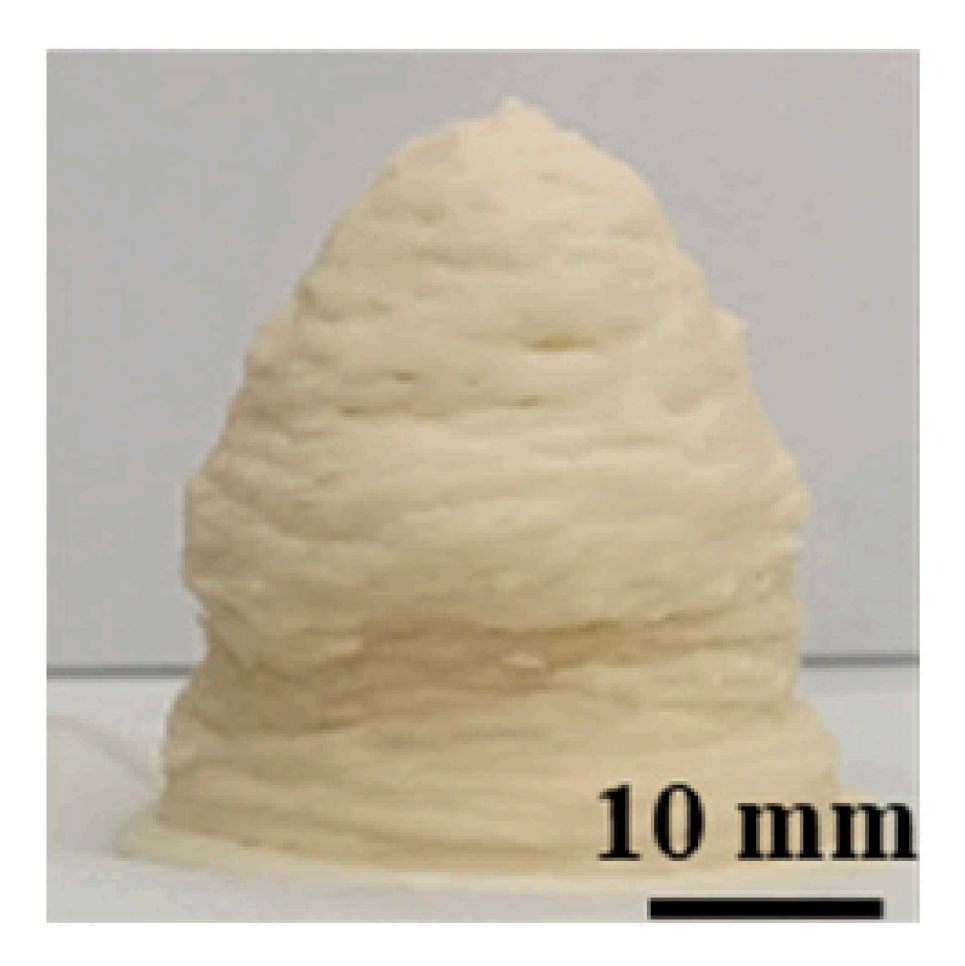	34.3 ± 0.5	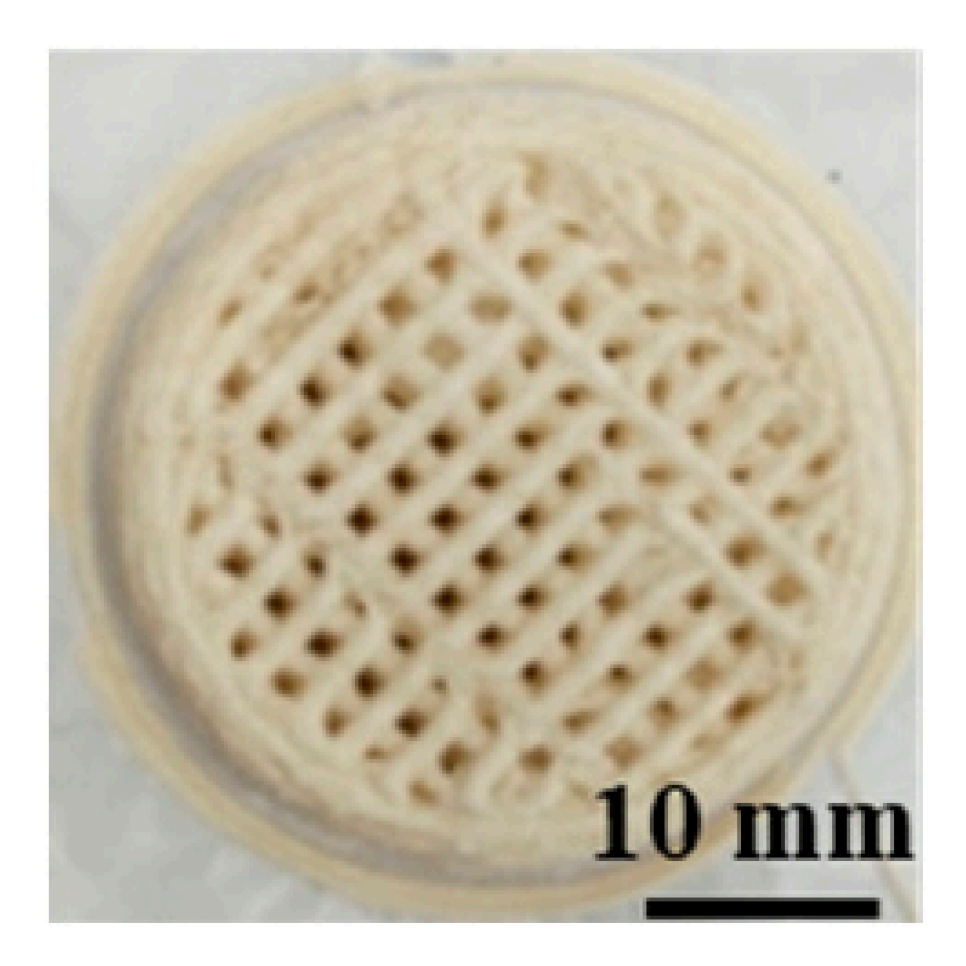	54.6 ± 1.0	4.0 ± 0.2	Blurred filament boundaries
C	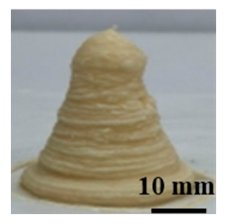	31.2 ± 0.3	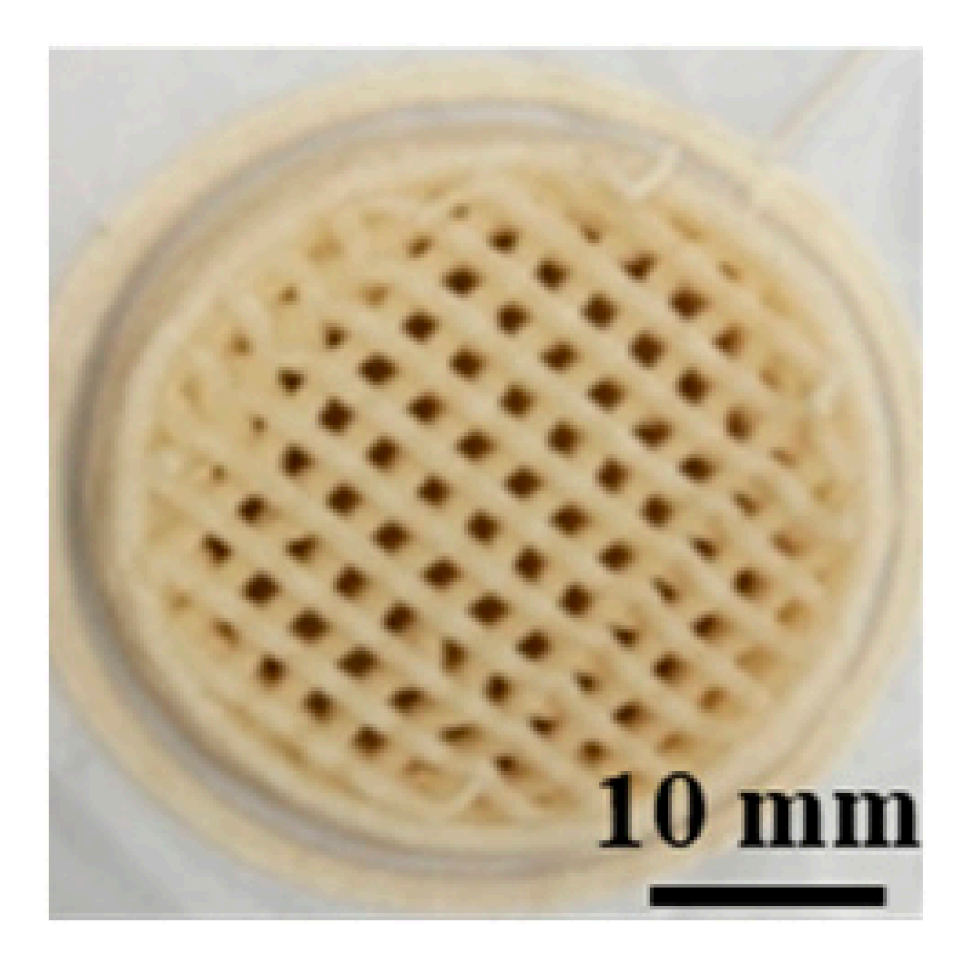	54.3 ± 2.1	4.6 ± 0.2	Good shape fidelity
D	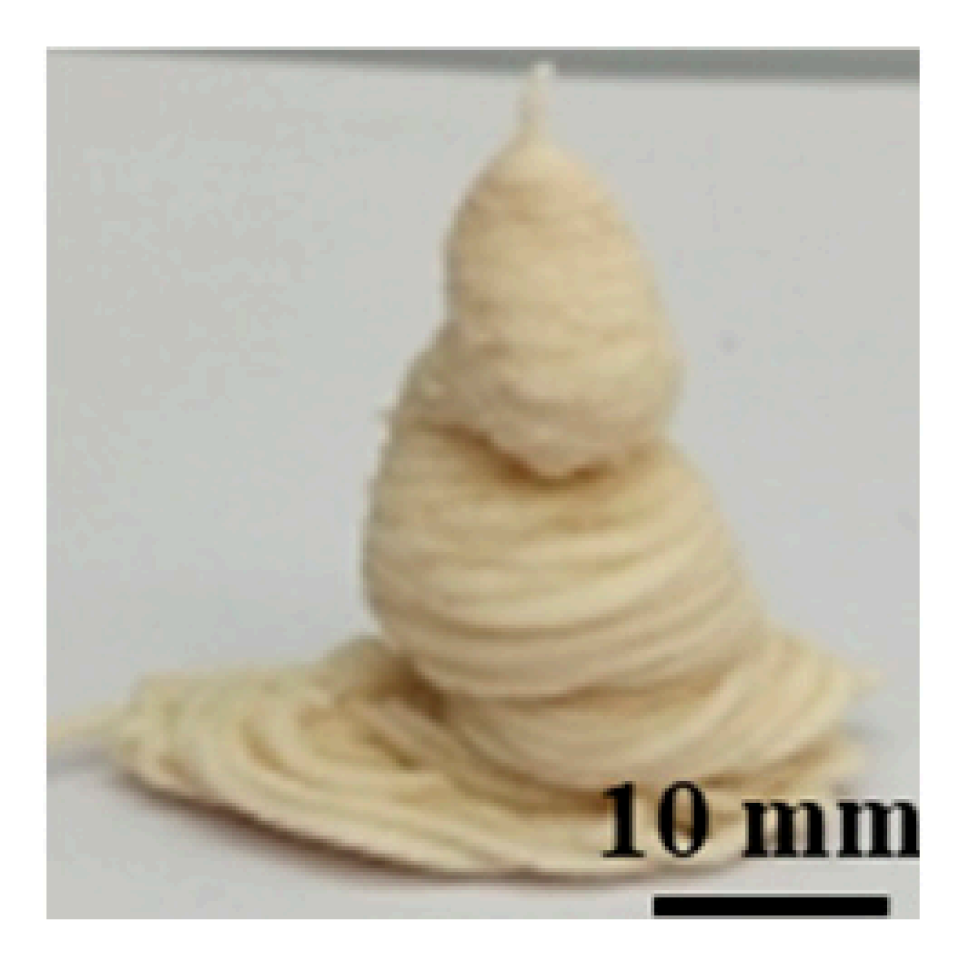	30.1 ± 0.6	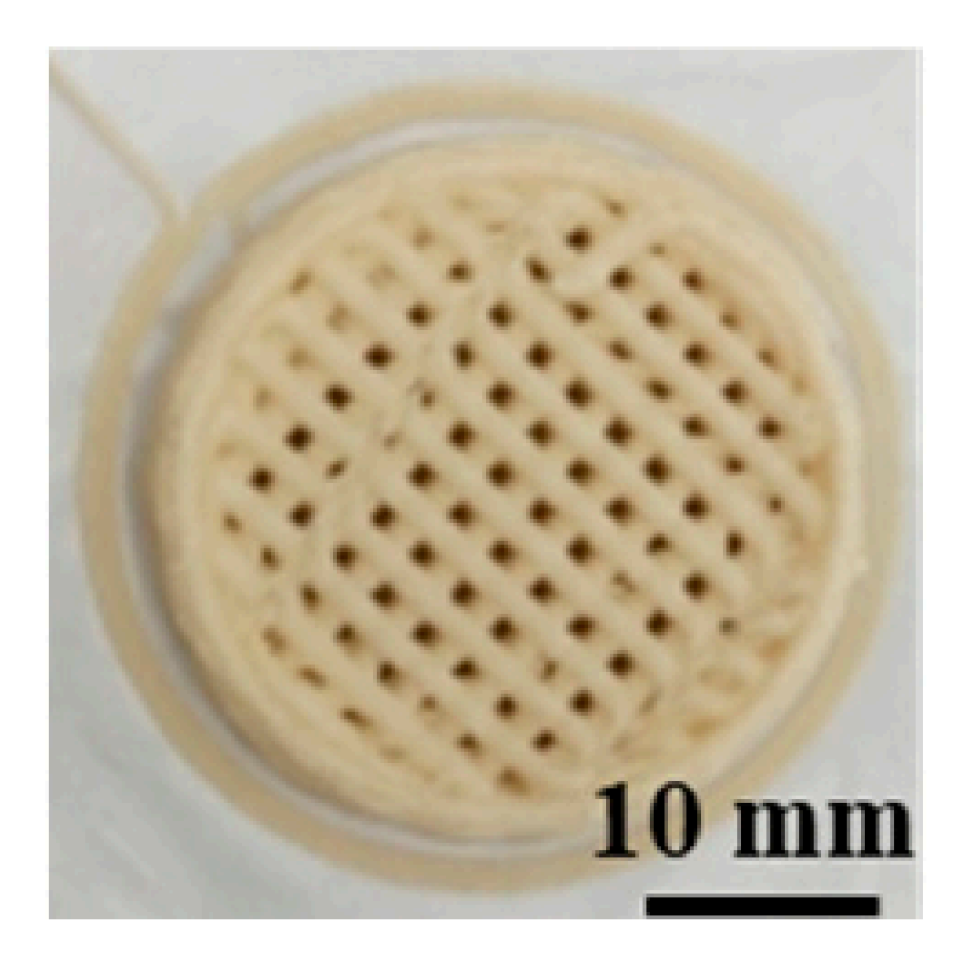	54.7 ± 1.0	3.9 ± 0.1	Blurred filament boundaries
E	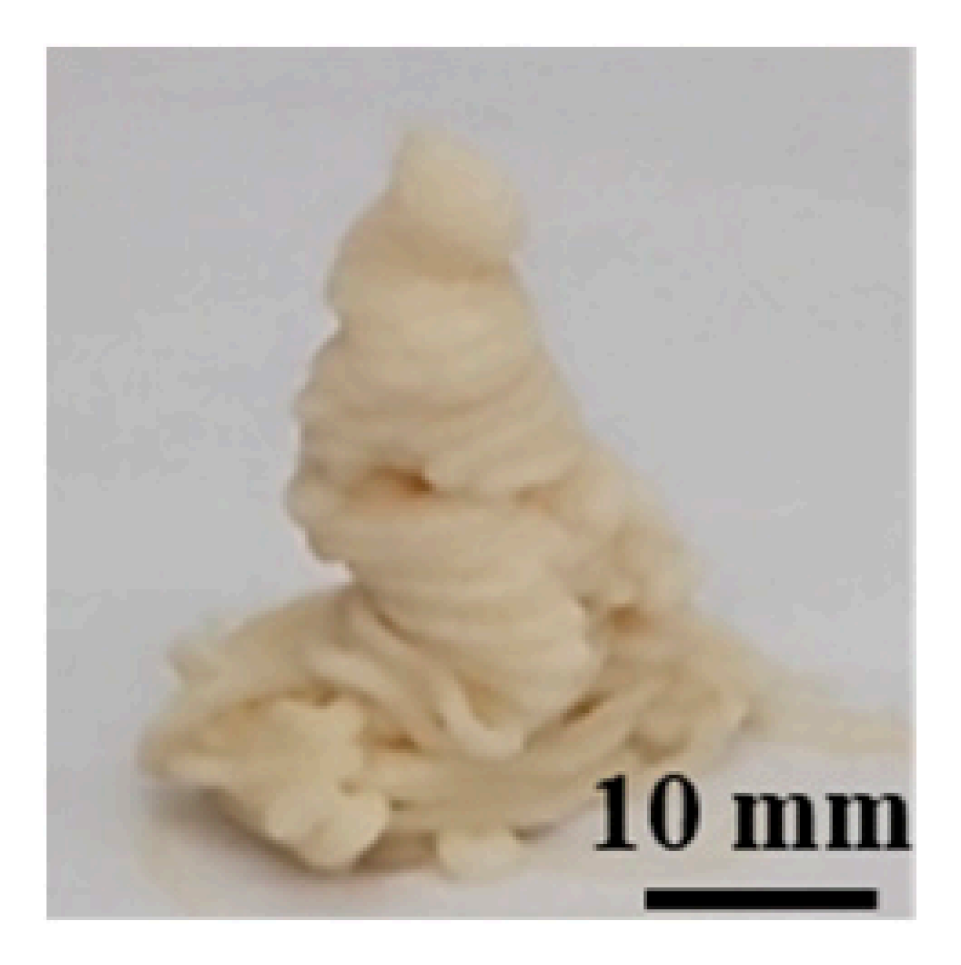	20.3 ± 0.3	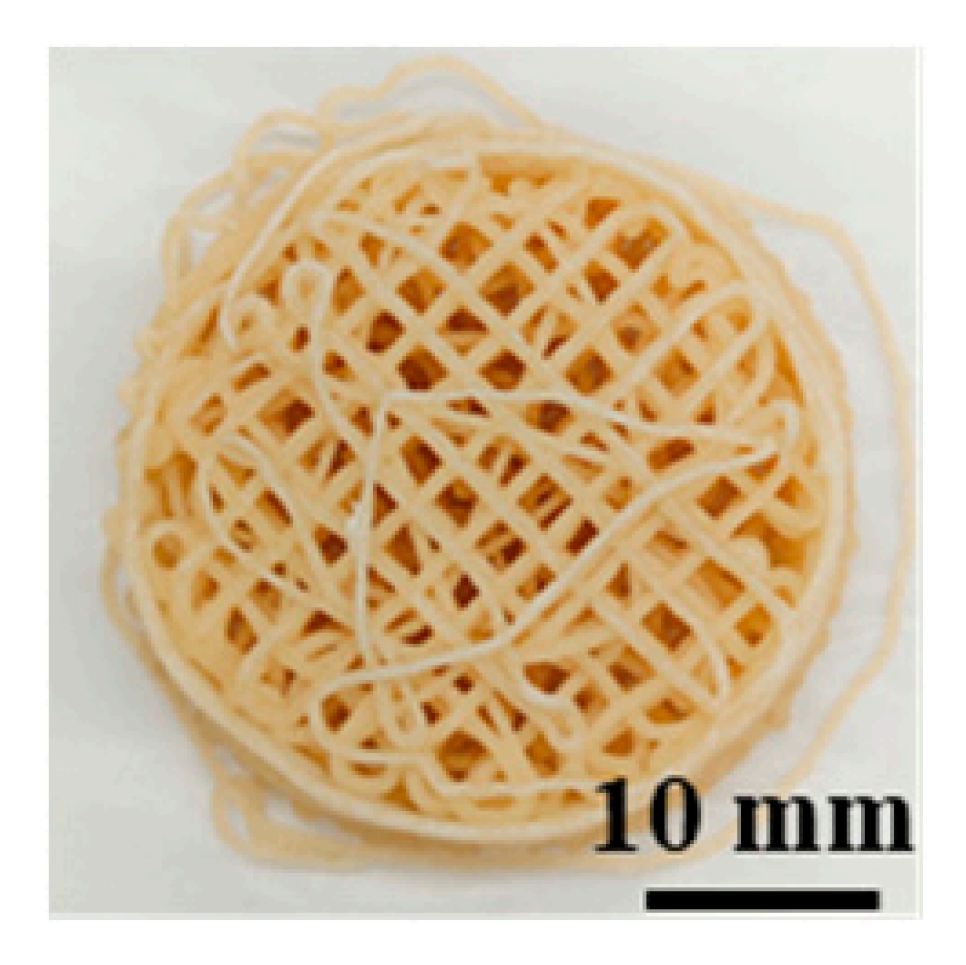	46.8 ± 3.2	3.1 ± 0.2	Failed to maintain the basic shape
F	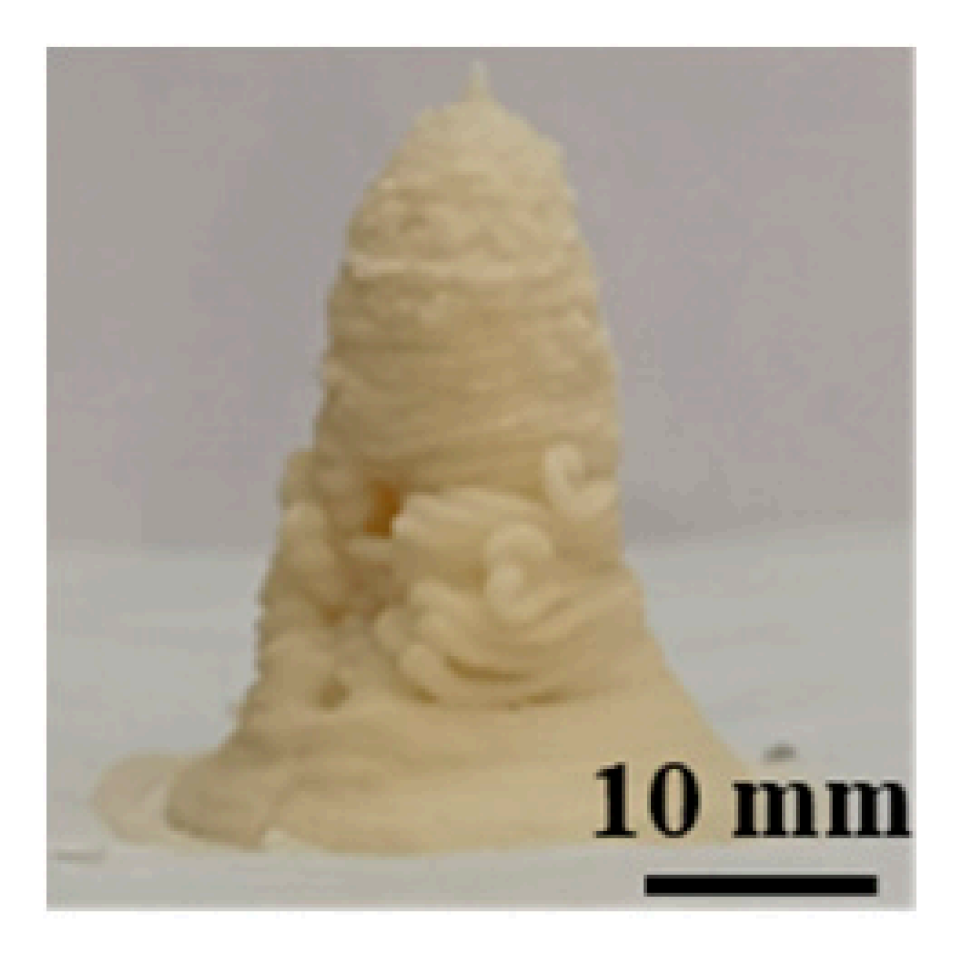	24.0 ± 0.4	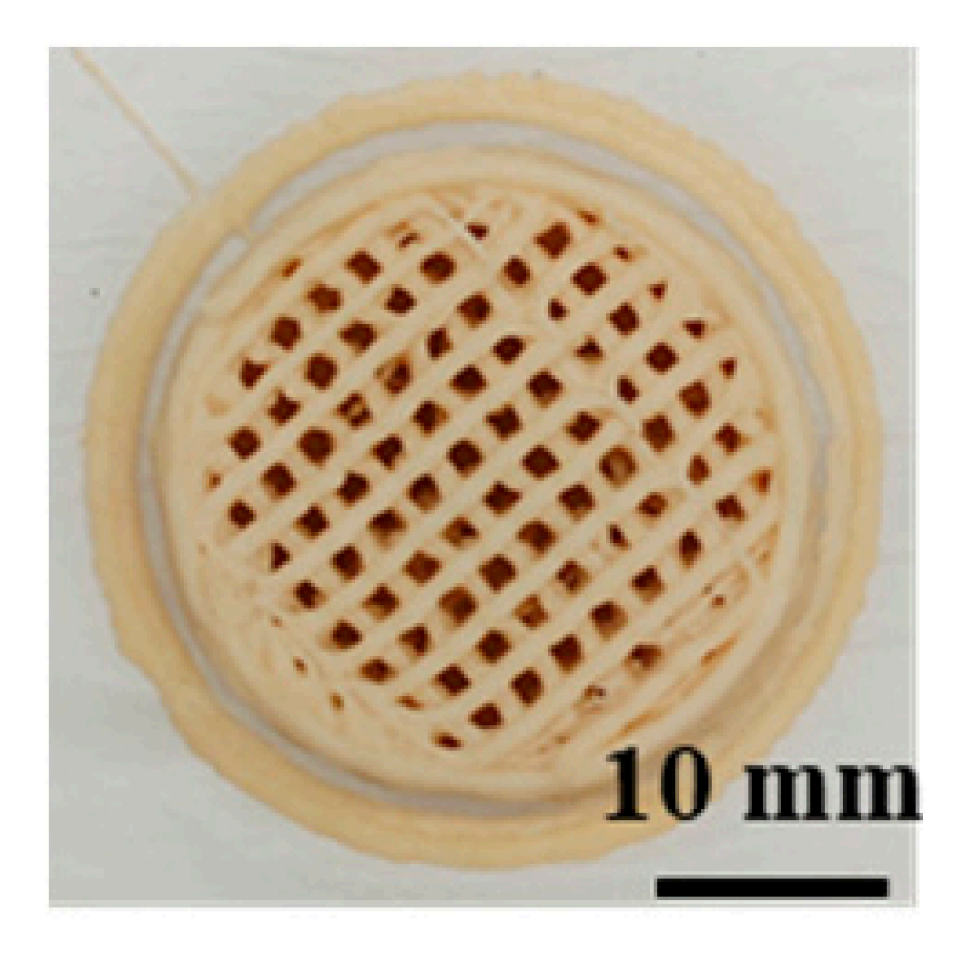	46.2 ± 1.0	4.3 ± 0.2	Good shape fidelity, but irregular filament arrangement
G	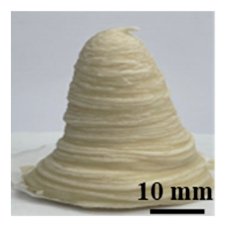	25.2 ± 0.2	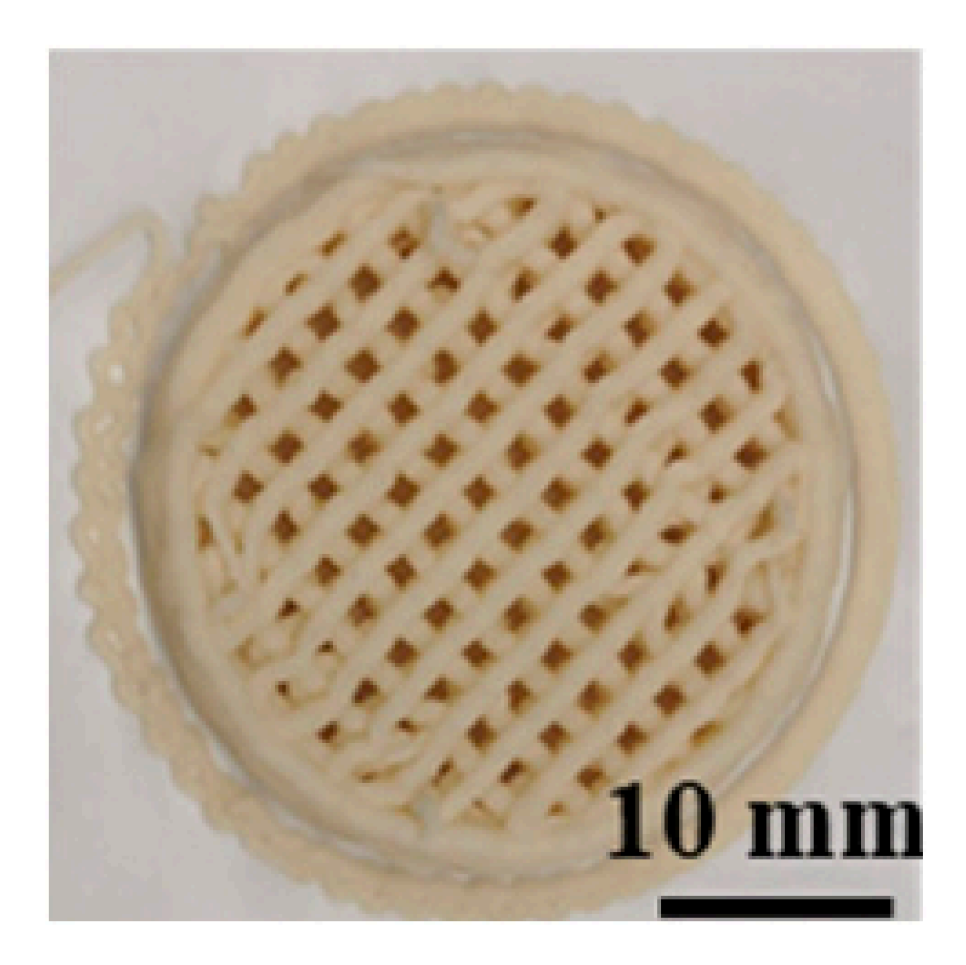	46.0 ± 1.5	4.5 ± 0.3	Good shape fidelity with slight filament blurring
H	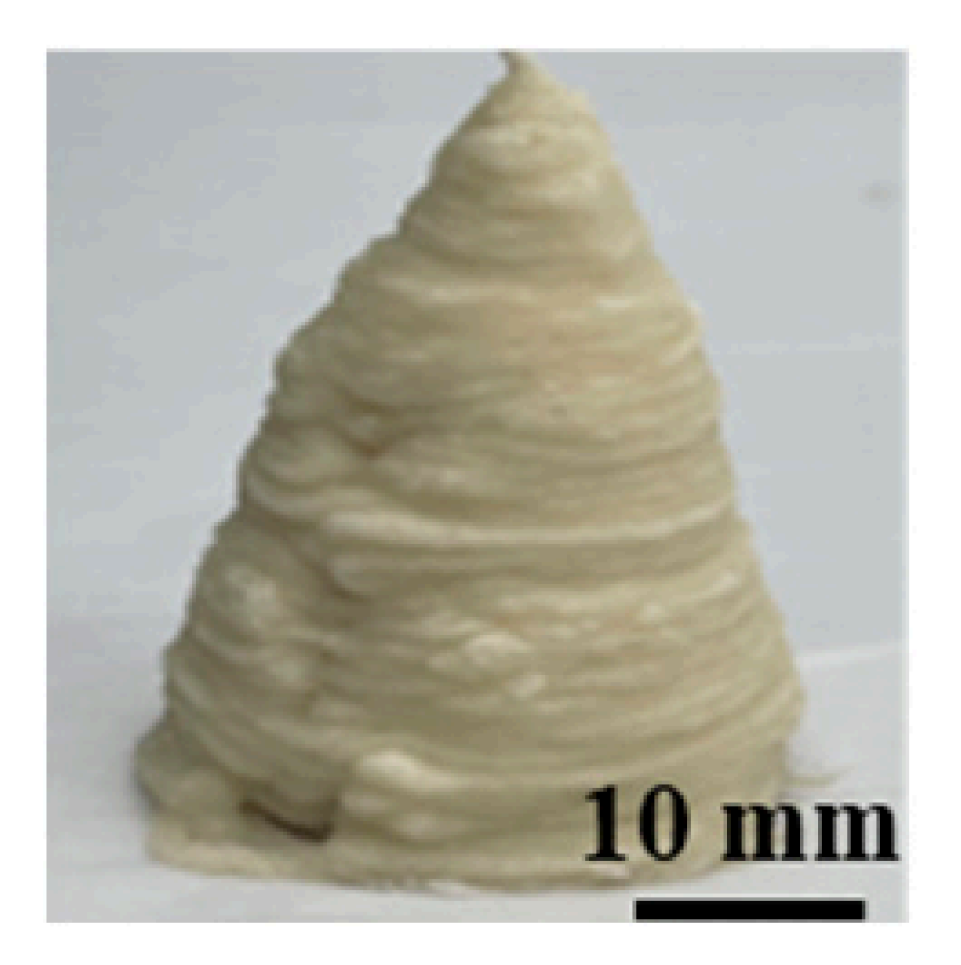	23.0 ± 0.4	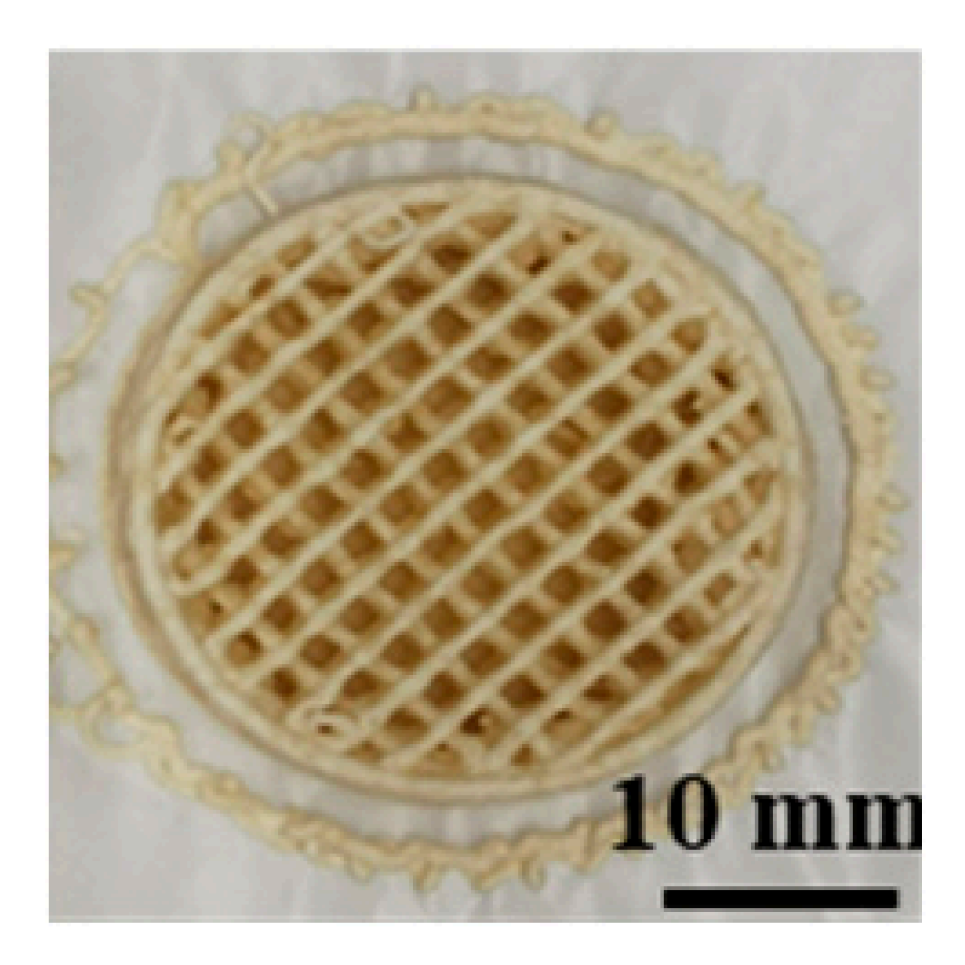	47.2 ± 2.0	4.2 ± 0.1	Good shape fidelity, but irregular filament arrangement

## Data Availability

The original contributions presented in the study are included in the article, further inquiries can be directed to the corresponding author.
